# How to improve users’ intentions to continued usage of shared bicycles: A mixed method approach

**DOI:** 10.1371/journal.pone.0229458

**Published:** 2020-02-24

**Authors:** Wang Zhanyou, Han Dongmei, Zhao Yaopei

**Affiliations:** 1 Office of Academic Research, Shandong Management University, Jinan, China; 2 School of Management Science and Engineering, Shandong University of Finance and Economics, Jinan, China; 3 School of Information Engineering, Shandong Management University, Jinan, China; 4 Party Committee Office, Shandong Management University, Jinan, China; The University of Tokyo, JAPAN

## Abstract

Sharing the use of a bicycle in China has changed people’s daily travel modes. Existing studies mainly explored the factors affecting individuals’ initial intentions to start using a shared bicycle, but few looked at the likelihood that a user would continue using one. Based on a post-acceptance model of information system (IS) continuance, this investigation proposed a research model to investigate factors influencing riders’ intentions to continued usage of shared bikes. Analysis involved structural equation modeling (SEM) and fuzzy-set qualitative comparative analysis (fsQCA) on data from 376 shared bicycle riders. The results from SEM showed that perceived usefulness, service quality, riders’ habits, overall satisfaction and the nature of the weather were the most important factors positively influencing users’ intentions to continue bike sharing. The results from fsQCA showed that six combinations of these variables were sufficient to explain continued usage. The conclusions of this study can be useful for operators to improve shared bicycle services.

## Introduction

In recent years, bicycle sharing has become immensely popular in China. According to the China Internet Network Information Center (CNNIC), as of June 2018, the number of users of shared bikes reached 245 million an increase of 24.32 million users in six months. The services are provided by bicycle operators on campuses, near subways or bus stations, residential or public service areas, and business districts using a time-sharing lease system. Sharing bicycles has brought great convenience to residents. However, from the perspective of market structure, the shared bike business represents a strongly competitive situation. The successful shared bike companies include Mobike and Hellobike, while twenty-two companies have gone bankrupt. The inconsistency of riders in continuing with a particular bike service is a primary reason that companies have gone out of business. Thus, investigating what determines the intentions of riders to discontinue using shared bikes or a specific company has become particularly urgent and important.

A review of the existing literature on bike sharing, showed that studies mainly focused on factors influencing the choice of bicycle [1, 2]; shared bike use behavior [3]; shared bike users’ subjective well-being [4]; the diffusion of public bike sharing systems [5]; the influence of bike sharing on other transportation modes such as car use [6]; the impact of weather conditions on bike use [7]; the effects of the built environment on bike sharing demand [8]; bike share rebalancing strategies, patterns, and purposes [9]; bike share safety [10]; bike share demand [11]; impact of pricing and transit disruptions on bike sharing [12]; optimization models for bike-sharing problems with transshipment [13]; bike share stations [14]; bike sharing travel patterns [15]; and, the shared bike users’ recommendations [16]. However, few studies have examined what controls the shared bike users’ intentions to continued or discontinued usage of shared bikes. What’s more, most existing studies are conducted from a single perspective, without comprehensive consideration of the impact of user, system and environmental factors on bike users’ intentions to continued usage of shared bikes. According to Ku, et al. [17], providing gratifying user experiences of the service is essential to motivate them to stay with it. Hong, et al. [18] and Chae, et al. [19] both showed that catering to users’ intentions to persist in using shared bikes was vitally important for the success of companies operating such a service.

To address this lack of research data, we adapted a post-acceptance model of information system continuance, to create a research model to investigate the factors motivating riders to continue using a shared bike service. Personal factors such as perceived usefulness, satisfaction with ease of use, travel distance, and influence of weather, together with operational factors such as equipment and service quality were considered in attempting to determine why riders will continue using a particular shared bike service. The paper is organized as follows. Section 2 describes the theoretical background and hypotheses. Section 3 outlines the research methods, and the results of SEM and fsQCA analyses are given in Section 4. Finally, the implications of the study and its contribution to the knowledge in the field are described in Section 5.

## Theoretical background

### A post-acceptance model of IS continuance

A post-acceptance model of IS continuance has been proposed by Bhattacherjee [20], in which perceived usefulness positively affected satisfaction, and satisfaction prompted users to continue using IS. This post-acceptance model of IS continuance has been widely adopted and extended [21–24]. For example, Choi, et al. [25] expanded the post-acceptance model to show that functional benefits, ease of use, and perceived enjoyment positively affected users’ satisfaction and trust, which contributed to the desire to continue using certain travel apps. Testing the IS continuance model, Liu, et al. [26] found that perceived ease of use positively affected user satisfaction. Some scholars also considered the influence of user habits on the post-acceptance model [27, 28]. For example, Shiau and Luo [29] added habit to the research model, and confirmed that habit positively affected users satisfaction, which promoted intentions to continue use. According to the post-acceptance model of IS continuance, perceived usefulness has positive effect on satisfaction, and finally satisfaction positively affects users’ IS continuance intention. Some scholars proved that perceived ease of use and habit positively affects user satisfaction in different contexts [25, 29]. Based on the above discussion, we proposed four hypotheses:

H1: Perceived usefulness positively affects users’ satisfaction.
H2: Perceived ease of use positively affects users’ satisfaction.
H3: Habit positively affects users’ satisfaction.
H4: Satisfaction positively affects continued usage intention.

### The relationship between system factors and satisfaction

After further research on the post-acceptance model of IS continuance, some scholars realized that service quality and system quality were also important factors influencing an individual’s decision to continue with a particular service [30, 31]. For example, Hsu, et al. [32] confirmed that the quality of the system, the resulting service and the information provided significantly affected user satisfaction. Lien et al. [33] found that service quality of WeChat was an important predictor of satisfaction, and the better the satisfaction the more likely a user was to stick with the same provider. Almarashdeh [34] found that both service quality and system quality positively affected the satisfaction of the users of a learning management system. In generally, improvements in service and system quality have been shown to be major drivers of user satisfaction in a variety of contexts [35, 36]. Thus, we also hold that the excellence of operation of bike sharing systems and the perceived quality of their service will have a positive effect on riders’ satisfaction. This study, therefore posits that:

H5: Service quality positively affects users’ satisfaction.
H6: System quality positively affects users’ satisfaction.

### The relationship between weather and ride distance on decision to continue service usage

In recent years, with the rise of bike sharing services, the factors affecting usage, especially environmental factors such as weather and travel distance, were also considered important influencers of users’ intentions to continue using shared bikes [8, 37–40]. In this study, the effect of weather was measured by the statements, “In the spring I will use bike-sharing more often” and “My use of bike sharing decreases in the winter”. Gebhart and Noland [7] confirmed that the likelihood of using a shared bike and the duration of trips was affected by cold, rain, and high humidity. Kim [41] showed evidence that temperatures over 30 °C reduced bicycle usage in general. This paper showed that bad weather can have a negative effect on riders’ use of a shared bike service. Ride distance is another factor affecting bicycle use [1]. In general, users are more likely to continue using shared bikes when travelling only short distances [40, 42], which means that travel distance negatively affects users’ continued usage intention. Thus, we propose the following hypotheses:

H5: Weather positively affects users’ continued usage intention.
H6: Travel distance negatively affects users’ continued usage intention.

**Fig 1:**
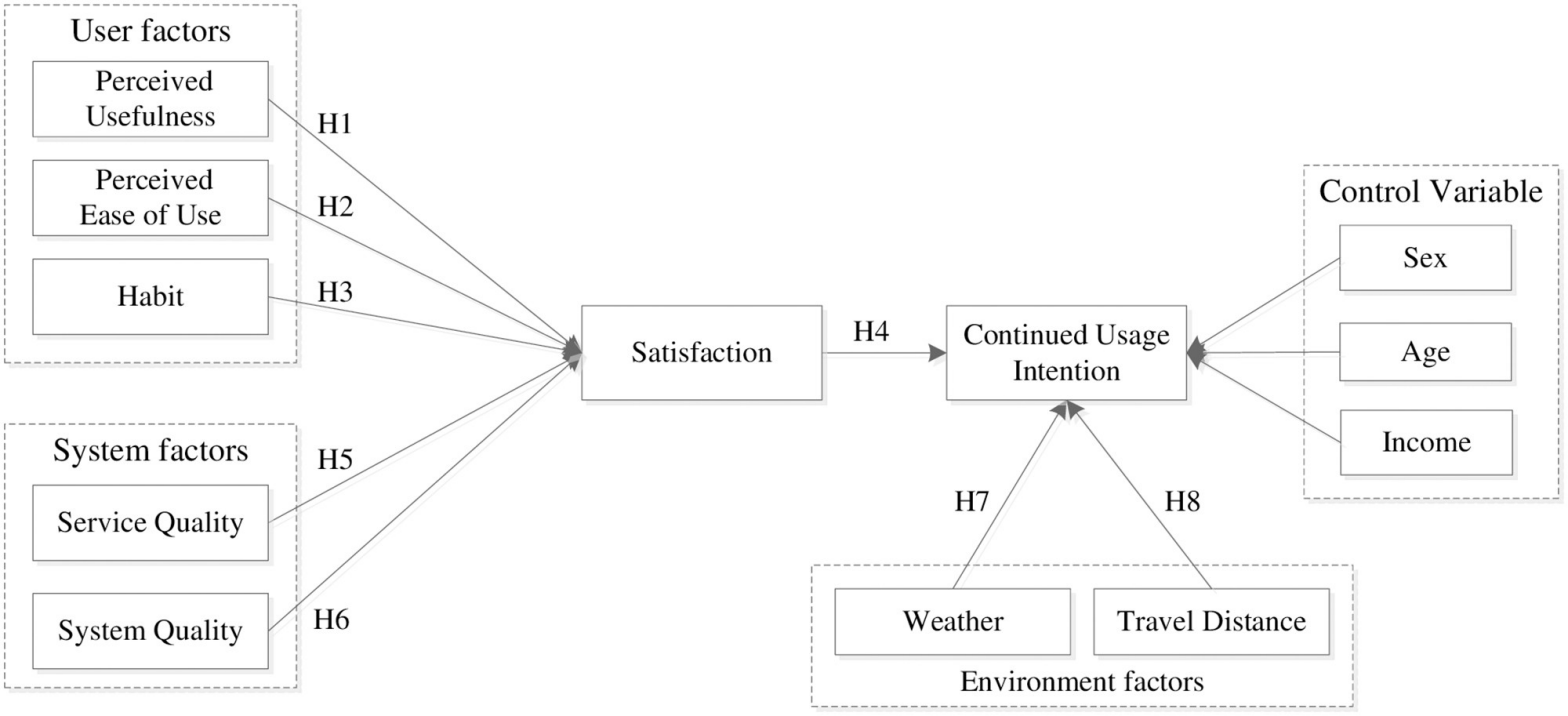
Factors influencing users’ intentions to continued usage of shared bicycles.

In addition, user sex, age, and monthly income were tested as mediating variables. Our research model can be seen in Fig 1. The shared bicycle service consists of both of the interface (apps) and the mobility/maintenance condition of bikes themselves. Accessibility, affordability, and the purpose of each trip are some of the multiple factors that typically influence the satisfaction of mobility. However, the Chinese shared bicycle service has its’ own characteristics. For example, the dockless and payment system efficiently merged into mobiles. Thus, this paper applies the ‘post-acceptance model of IS continuance’ to the usability of (mobile) apps. This is a precondition of our model.

## Materials and methods

### Variables and measures

The questionnaire method was used to validate the conceptual model (Table 1). All of the measured items were taken from other published studies: service quality was adapted from Oghuma, et al. [43]; system quality was from Chen [44]; perceived usefulness and perceived ease of use were taken from Ma, et al. [4]; habit was adapted from Xin, et al. [2]; customer satisfaction was derived from Zhang, et al. [45]; and, finally, the measures of continued usage intention were adapted from Bhattacherjee [20]. The questionnaires gauged responses on a 7-point Likert scale. The influence of weather was measured by the statement “In the spring I will use shared bikes more often” and “My use of bike-sharing decreases in the winter”. Travel distance was measured by the question “How far do you travel on a shared bike?” and the answer choices were: 1 to 2 kilometers, 2–3 kilometers, 3–4 kilometers, 5 kilometers and above.

**Table 1:** Questionnaire statements for measuring usage factors.

Factors	Statements
Service Quality (SQ)	The functions and services provided by the shared bike provider was comfortable
	When we face service and system problems, the shared bike provider provides services with sincere attitude
	The shared bike provider provide accurate and reliable information
	The shared bike provider gives me prompt services
	The shared bike provider gives the right solution to my request during service and system failures
System Quality (XQ)	I expect that the system of the shared bike provides good access.
	I expect that the system of the shared bike is responsive to members’ requests.
Perceived Usefulness (PU)	Using the shared bike could make my travel more convenient
	Using the shared bike could make my travel more efficient
	I find the shared bike to be useful to my daily travel
Perceived Ease of Use (PE)	My interaction with the shared bike is easy and understandable
	My interaction with the facilities and services of shared bike is easy and understandable
	The shared bike is easy for me to use
Habit (HA)	Shared bicycle has become a natural choice for me to travel at a short distance.
	When I travel at short distances, use of a shared bicycle comes to my mind
	Shared bicycle has become a spontaneous short distance travel tool to me.
Customer satisfaction(CS)	I feel good regarding my decision to riding a shared bike for travel.
	I think that ride a shared bike for travel is a good idea
	Overall, I am satisfied with the experience of riding a shared bike for travel
Continued usage intention(CI)	I intend to continue using shared bike rather than discontinue its use.
	My intentions are to continue using shared bike than use any alternative means.
	If I could, I would like to discontinue my use of shared bike (reverse coded).

### Data collection

We designed the questionnaire on a survey platform (Soujump.com), sent links to the WeChat group and collected the questionnaires by the snowball method. WeChat is the most popular social network in China. According to Tencent, WeChat has 1.11 billion users as of April 2019 [46]. WeChat users form a WeChat group according to similar interests or a specific purpose. WeChat group is a group of people who can chat with each other. Users can invite friends or people with shared interests to chat with each other in a group. Almost everyone has a WeChat group. We recruited our participants from WeChat groups.

This study used the snowball method to collect the data. Since bike-share users are difficult to sample with probability methods, this study used a non-probability method. Samples used in this study is a non-probability sample. The author started the snowball recruitment method. This study recruited participants based on the criteria of having used shared bikes and continue to use them. The author first shared the questionnaire link in a WeChat group. The members of this group are university students and colleagues at Shandong Management University. The shared bike was first introduced on college campuses, and university students are a major user of shared bikes. Thus, we have reason to believe that many people in the group that we choose use shared bikes. After the initial recruitment through WeChat, in order to cover more areas and engage more people, ten teachers were recruited to fill out the questionnaire and asked to share the questionnaire link with their WeChat groups. These teachers are college teachers that the author knew. The author had their WeChat account numbers and recruited them on WeChat as additional seeds. The teachers were from Tsinghua University, Shandong Agricultural University, Qilu University of Technology, Linyi University, Shandong Agriculture and Engineering University, Weifang Engineering Vocational College, Liaocheng Vocational College, Binzhou University, Fujian Academy of Social Sciences, and Shandong Technology and Business University in China. The members of these WeChat group are mainly university students and university colleagues.

We sent the questionnaires from March 23 to March 25, 2019. 2,540 WeChat group users were invited to take part in the survey; 483 completed the survey, and out of these, we rejected 27 because the respondents had never used shared bikes, and we excluded 80 for inconsistencies according to the contrary measurement questions. We used the remaining 376 valid questionnaires (77.8%) in this study. The survey participation rates were calculated using the standards published by the American Association for Public Opinion Research (AAPOR). The sampling process yielded a raw participation rate of 14.8% (RR4, AAPOR) [47]. In order to determine whether the sample size is sufficient, we use Daniel Soper’s a priori sample size calculator for structural equation models [48]. The result shows that the minimum required sample size is 256 for this study. Thus, our sample size is larger than the required value. We have written informed consent from all participants, and there are no minor participants. The Shandong Management University Ethics Committee approved the protocol and informed consent forms for this study. According to research report on China’s Shared bike market in the first quarter of 2019 conducted by Big Data Research, as of April 2019, the age distribution of bike-sharing users is as follows: 77.4% are young people under 35 years old, among which 19.2% are under 25 years old, 25.4% are between 25 and 35 years old, and 32.8% are between 31 and 35 years old [49]. Our sample’s age distribution is consistent with the results of Big Data Research. Thus, our sample can be representative of the population of interest. The respondents’ characteristics can be seen in Table 2.

**Table 2:** Respondents’ characteristics.

Demographic variables		Number	%
Gender	Male	133	35.37
	Female	243	64.63
Age	<=20 years old	101	26.86
	21–30 years old	120	31.91
	31–40 years old	122	32.45
	40–50 years old	28	7.45
	>50 years old	5	1.33
Education	Senior middle school or below	13	3.46
	Junior college	46	12.23
	Bachelor’s degree	188	50.00
	Master’s degree or above	129	34.31
Income	<=3,000	171	45.48
	3,001–5,000	55	14.63
	5,001–8,000	92	24.47
	> 8,001	58	15.43
Brand of shared bike	OFO	69	18.35
	Mobike	108	28.72
	Hellobike	147	39.10
	Others	52	13.83
Use frequency	More than once a day	12	3.19
	2–3 times a week	76	20.21
	2–3 times one month	143	38.03
	Less than 10 times a year	145	38.56
Travel distance	1 kilometer and below	94	25.00
	1–2 kilometer	134	35.64
	2–3 kilometers	76	20.21
	3–4 kilometers	35	9.31
	5 kilometers and above	37	9.84
Time of using a shared bicycle	1 months and below	75	19.95
	1–3 months	34	9.04
	3–6 months	26	6.91
	6–12 months	24	6.38
	1 years and above	217	57.71
Purpose of using a shared bicycle	Go for work	61	16.22
	Daily walking	204	54.26
	Recreation & Entertainment	148	39.36
	Shopping	60	15.96
	Change to other means of transportation	135	35.90
	Others	52	13.83
Reasons for choosing a shared bike	Convenient	295	78.46
	Save time	206	54.79
	Exercise	130	34.57
	Save cost	137	36.44
	Low carbon for environmental protection	177	47.07
Other transportation vehicles at home	Car	215	57.18
	Bicycle	19	5.05
	A storage battery car	85	22.61
	Motorcycle	4	1.06
	Others	53	14.10

### Measurement model

The testing of the model showed that it had good reliability and validity (Table 3). Cronbach’s alpha was greater than 0.7, composite reliability was larger than 0.7, and average variance extracted (AVE) was greater than 0.5. [50]. Moreover, the scale had good discriminant validity because the square root of the AVE of the measured variables was greater than their correlation coefficients. Table 4 shows that all measurement variables and potential variables displayed high correlation coefficients, while correlation coefficients of other latent variables were relatively low, indicating that the measurement items were internally consistent and had good distinction capability [51].

**Table 3:** Descriptive statistics and inter-construct correlations.

Item	**Cronbach’s Alpha**	**CR**	**AVE**	**SQ**	**XQ**	**PU**	**PE**	**HA**	**TD**	**WE**	**CS**	**CI**
**SQ**	0.886	0.917	0.689	0.830								
**XQ**	0.870	0.939	0.884	0.751	0.940							
**PU**	0.809	0.886	0.721	0.498	0.395	0.849						
**PE**	0.874	0.921	0.796	0.507	0.459	0.536	0.892					
**HA**	0.898	0.936	0.830	0.564	0.484	0.581	0.467	0.911				
**TD**	1.000	1.000	1.000	0.098	0.047	0.063	0.100	0.150	1.000			
**WE**	0.632	0.833	0.716	0.360	0.329	0.402	0.332	0.502	0.196	0.846		
**CS**	0.876	0.924	0.802	0.603	0.516	0.677	0.520	0.737	0.119	0.474	0.896	
**CI**	0.784	0.901	0.820	0.623	0.503	0.614	0.527	0.732	0.165	0.453	0.809	0.906

Service Quality (SQ); System Quality (XQ); Perceived Usefulness (PU); Perceived Ease of Use (PE); Habit (HA); Travel Distance (TD); Weather (WE); Satisfaction(CS); Continued usage Intention(CI).

**Table 4:** Cross loadings.

Item	SQ	XQ	PU	PE	HA	TD	WE	CS	CI
**SQ1**	**0.761**	0.506	0.496	0.496	0.512	0.105	0.257	0.562	0.582
**SQ2**	**0.844**	0.636	0.364	0.343	0.460	0.050	0.298	0.467	0.491
**SQ3**	**0.810**	0.619	0.367	0.446	0.453	0.123	0.273	0.440	0.500
**SQ4**	**0.873**	0.656	0.421	0.447	0.471	0.061	0.350	0.515	0.499
**SQ5**	**0.856**	0.704	0.394	0.354	0.430	0.069	0.315	0.495	0.494
**XQ1**	0.668	**0.947**	0.383	0.443	0.456	0.060	0.331	0.509	0.497
**XQ2**	0.749	**0.934**	0.359	0.419	0.455	0.028	0.286	0.459	0.446
**PU1**	0.350	0.287	**0.817**	0.377	0.408	0.001	0.300	0.496	0.442
**PU2**	0.415	0.349	**0.874**	0.458	0.410	0.028	0.309	0.533	0.462
**PU3**	0.485	0.361	**0.856**	0.513	0.624	0.112	0.399	0.668	0.629
**PE1**	0.393	0.349	0.429	**0.893**	0.370	0.047	0.285	0.397	0.424
**PE2**	0.472	0.428	0.447	**0.894**	0.411	0.099	0.256	0.409	0.452
**PE3**	0.481	0.439	0.536	**0.890**	0.453	0.113	0.334	0.552	0.517
**HA1**	0.483	0.419	0.531	0.457	**0.895**	0.188	0.448	0.623	0.626
**HA2**	0.535	0.450	0.501	0.404	**0.913**	0.107	0.456	0.677	0.680
**HA3**	0.521	0.453	0.556	0.417	**0.925**	0.122	0.469	0.709	0.692
**TD**	0.098	0.047	0.063	0.100	0.150	**1.000**	0.196	0.119	0.165
**WE1**	0.371	0.364	0.450	0.354	0.548	0.193	**0.934**	0.492	0.502
**WE2**	0.206	0.141	0.163	0.167	0.230	0.128	**0.748**	0.265	0.192
**CS1**	0.484	0.446	0.626	0.465	0.711	0.122	0.456	**0.915**	0.719
**CS2**	0.499	0.438	0.622	0.460	0.662	0.072	0.425	**0.928**	0.749
**CS3**	0.642	0.505	0.568	0.473	0.604	0.127	0.392	**0.842**	0.704
**CI1**	0.583	0.461	0.588	0.530	0.700	0.132	0.467	0.816	**0.929**
**CI2**	0.543	0.451	0.518	0.413	0.620	0.172	0.340	0.630	**0.881**

## Results

### Hypothesis testing using the structural model

**Fig 2:**
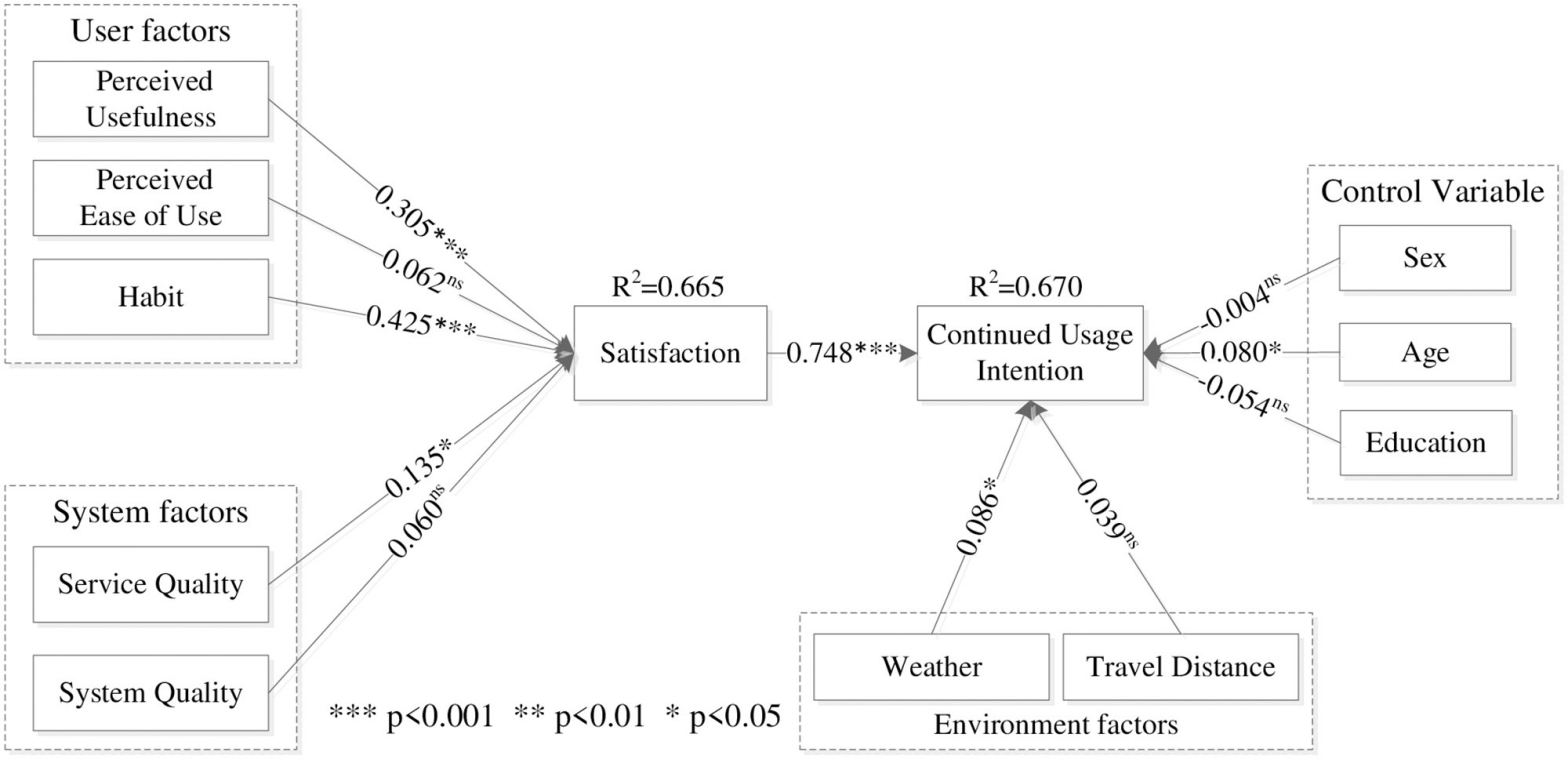
Results of structural model analysis: Pathways to shared bicycle use behavior through user factors, system factors, satisfaction, and environment factors.

Fig 2 shows that perceived usefulness had a positive effect on user satisfaction (β = 0.305, t = 6.109), confirming H1. Perceived ease of use did not have a positive effect on user satisfaction (β = 0.062, t = 1.115), thus, H2 was not supported. Habit had a positive effect on user satisfaction (β = 0.425, t = 8.600), confirming H3. The impact of users’ satisfaction on their intention to continue usage was significant (β = 0.748, t = 21.715), confirming H4. Service quality had a positive effect on user satisfaction (β = 0.135, t = 2.471), confirming H5. However, system quality did not have a positive effect on user satisfaction (β = 0.060, t = 1.225), thus, H6 was not supported. Finally, weather had a positive effect on riders’ intentions to continued usage (β = 0.086, t = 2.124), thus, H7 was supported. Travel distance did not have a positive effect on users’ intentions to continued usage (β = 0.039, t = 1.387), thus, H8 was not supported. The results of analyzing control variables showed that sex did not have a negative effect on riders’ intentions to continue with the service (β = -0.004, t = 0.142), while age had a positive effect on user intentions (β = 0.080, t = 2.373) and education did not (β = -0.054, t = 1.613).

### Measurement invariance across gender

Measurement invariance analysis was conducted to investigate factor structure similarity across gender [52]. The study divided the groups into two: groups 1 and 2, as male and female groups, respectively. Using Amos 20.0, multi-group confirmatory factor analysis was used to test measurement invariance (MI). According to Petrowski, et al. [53], configural, metric, and scalar invariance are enough for implementing measurement invariance. For tests of invariance, the changes of CFI (ΔCFI) were used as indices, and ΔCFI ≤ 0.01 indicate strong invariance [54].

First, the results in Table 5 show that the configural invariance model, which simultaneously estimates all model parameters freed across groups results in an excellent model fit (CFI = 0.914, RMSEA = 0.059). Second, weak invariance constraints on all factor loadings are invariant across gender groups. The results show that ΔCFI were below the cut-off recommended by Ren, et al. [54]. The model fit was excellent to good (CFI = 0.914; RMSEA = 0.058). Third, strong invariance constraints on all items intercepts are invariant across groups. The results show that ΔCFI were below the cut-off recommended by Ren, et al. [54]. The model fit was excellent to good (CFI = 0.907; RMSEA = 0.058). Finally, strict invariance constraints on all items residual variances are invariant across groups. The results show that the ΔCFI were below the cut-off recommended by Ren, et al. [54]. The model fit was excellent to good (CFI = 0.904; RMSEA = 0.057). In summary, the measurement invariance between male and female was demonstrated.

**Table 5:** The analysis of factorial invariance for gender using multi-group confirmatory factor analysis.

Model	Test	χ^2^	Df	CFI	ΔCFI	RMSEA	ΔRMSEA	MI Test ^a^
Model 0	Configural invariance	928.3	404	0.914		0.059		Y
Model 1	Weak invariance	948.6	419	0.914	0.000	0.058	-0.001	Y
Model 2	Strong invariance	1025.0	455	0.907	-0.007	0.058	0.000	Y
Model 3	Strict invariance	1067.4	478	0.904	-0.003	0.057	-0.001	Y

df = degree of freedom; CFI = robust version of the Comparative Fit Index; ΔCFI = differences between models (0 and 1, 1 and 2, and 2 and 3) in robust CFI; RMSEA = robust version of the root mean square of approximation; ΔRMSEA = differences between models (0 and 1, 1 and 2, and 2 and 3) in robust RMSEA;

^a^ = ΔCFI ≤ 0.010 supplemented by ΔRMSEA ≥ -0.015 indicates non-invariance.

Y marks invariance.

### Fuzzy-set qualitative comparative analysis (fsQCA)

Before using fsQCA to analyze the same data set on which SEM was used, it was necessary to calibrate the scales [55, 56]. This was done by calculating the mean score of each construct, identifying three fuzzy conversion metrics as full membership (1), cross-over point (0.5) and full non-membership (0), and finally transferring the original data to continuous data from 0 to 1 through calculating scalars and log adds [57]. Afterwards, we examined whether each conditional variable was necessary or sufficient for the resultant variable [58]. After applying the necessary conditions test (Table 6), we determined that perceived usefulness, perceived ease of use, weather, and satisfaction were all necessary conditions to ensure that shared bike riders continued to use the service because all consistency values were greater than 0.90 [57].

**Table 6:** Necessary conditions from fsQCA.

Items	Continued usage Intention	
	consistency	coverage
Service Quality	0.870633	0.919970
~ Service Quality	0.408161	0.850953
System Quality	0.837713	0.913520
~ System Quality	0.441233	0.866850
Perceived Usefulness	**0.963022**	0.841100
~ Perceived Usefulness	0.250085	0.889757
Perceived Ease of Use	**0.972845**	0.818291
~ Perceived Ease of Use	0.206963	0.872701
Habit	0.884286	0.930444
~ Habit	0.378124	0.794993
Weather	**0.929874**	0.846440
~ Weather	0.291842	0.891243
Travel Distance	0.312436	0.930742
~ Travel Distance	0.860469	0.789175
Satisfaction	**0.965828**	0.898715
~ Satisfaction	0.287822	0.819192

Regarding the sufficiency conditions, the frequency cutoff in the truth table was set at 1 and the consistency cutoff at 0.945307 (all variables were present for the occurrence of continued usage intention). Six combinations of causal conditions in the intermediate solution (Table 7) could promote continued usage intention. The six sufficiency conditions combinations were service quality × perceived usefulness × perceived ease of use × weather × satisfaction (raw coverage: 0.79937; consistency: 0.961981); ~service quality × ~system quality × perceived usefulness × perceived ease of use × weather × ~travel distance (raw coverage: 0.331892; consistency: 0.958593); ~system quality × perceived ease of use ×habit × weather × ~travel distance × satisfaction (raw coverage: 0.367353; consistency: 0.987461); service quality × system quality × perceived ease of use × habit × weather × satisfaction (raw coverage: 0.712329; consistency: 0.982168); system quality × perceived usefulness × perceived ease of use × habit × weather × satisfaction (raw coverage: 0.726058; consistency: 0.976585); service quality × system quality × perceived usefulness × perceived ease of use × habit × ~travel distance × satisfaction (raw coverage: 0.657602; consistency: 0.982324). The most important condition combinations were service quality × perceived usefulness × perceived ease of use × weather × satisfaction and system quality × perceived usefulness × perceived ease of use × habit × weather × satisfaction because they present the highest raw coverage values.

**Table 7:** Intermediate solution.

Frequency cutoff: 1; consistency cutoff: 0.945307; all variables are present	Raw coverage	Unique coverage	Consistency
SQ*PU*PE*WE*CS	0.79937	0.0553346	0.961981
~SQ*~XQ*PU*PE*WE*~TD	0.331892	0.00242728	0.958593
~XQ*PE*HA*WE*~TD*CS	0.367353	0.00432336	0.987461
SQ*XQ*PE*HA*WE*CS	0.712329	0.00773698	0.982168
XQ*PU*PE*HA*WE*CS	0.726058	0.0139568	0.976585
SQ*XQ*PU*PE*HA*~TD*CS	0.657602	0.0213525	0.982324

solution coverage: 0.882959; solution consistency: 0.923336

Service Quality (SQ); System Quality (XQ); Perceived Usefulness (PU); Perceived Ease of Use (PE); Habit (HA); Travel Distance (TD); Weather (WE); Satisfaction(CS); Continued usage Intention(CI).

This study also conducted a bi-direction analysis that studied causal conditions in the intermediate solution (Table 8) that could promote discontinued usage intention. Regarding the sufficiency conditions, we set the frequency cutoff in the truth table at 1 and the consistency cutoff at 0.806066 (all variables were present for the occurrence of discontinued usage intention). Six combinations of causal conditions in the intermediate solution could promote discontinued usage intention. The most important condition combinations were satisfaction × weather × ~ habit × perceived ease of use × perceived usefulness × service quality (raw coverage: 0.646399; consistency: 0.793987) because they present the highest raw coverage values.

**Table 8:** Intermediate solution.

Frequency cutoff: 1; consistency cutoff: 0.806066; all variables are present	Raw coverage	Unique coverage	Consistency
CS*WE*~HA*PE*PU*SQ	0.646399	0.042731	0.793987
CS*~TD*~HA*PE*PU*~XQ*~SQ	0.574199	0.012908	0.895210
~CS*~TD*WE*PE*PU*~XQ*~SQ	0.543219	0.014689	0.944874
~CS*~TD*WE*~HA*PE*XQ*SQ	0.556840	0.008190	0.945149
~CS*~TD*~HA*PE*PU*XQ*SQ	0.560133	0.006944	0.946450
CS*WE*HA*PE*~PU*XQ*SQ	0.449033	0.003205	0.871458

solution coverage: 0.812517; solution consistency: 0.728937

Service Quality (SQ); System Quality (XQ); Perceived Usefulness (PU); Perceived Ease of Use (PE); Habit (HA); Travel Distance (TD); Weather (WE); Satisfaction(CS); Continued usage Intention(CI).

## Conclusions and implications

### Key findings

Based on a post-acceptance model of IS continuance, this paper investigated the factors potentially influencing the decisions of shared bike riders to continue using the shared bike services in China. Using both SEM and fsQCA analytical methods, data from 376 shared bicycle users were explored by means of a range of factors related to users’ preferences, system operability, weather and travel conditions that affect a rider’s intentions to stick with using shared bikes. This study did not study share bike users’ actual behavior as a technology acceptance model, and the theory of planned behavior indicates that users’ intention is an important predictor of users’ actual behavior. According to Si, et al. [59], sustainable usage intention of shared bike positively affects users’ sustainable usage behavior (β = 0.334, t = 9.001). This study provides some interesting revelations and allows us to draw valuable conclusions about shared bike usage.

Firstly, SEM results showed that users’ factors, such as perceived usefulness, and users’ habits significantly affect users’ satisfaction, both of which had positive effects on bike sharing users’ intentions to continued usage. However, the effect of perceived ease of use on users’ satisfaction was not significant. The conclusions of this study basically agree with those of Almarashdeh [34], who proved that perceived usefulness and service quality had the largest impact on satisfaction when using a learning management system, while the effect of perceived ease of use on users’ satisfaction was not significant. Prior research showed that users’ satisfaction was an important factor affecting users’ continued usage intentions in knowledge sharing [60]. Lin, et al. [61] found that perceived usefulness and users’ satisfaction significantly affected the intentions of users of social networking sites to persist in using them. Some scholars also pointed out that habit affects users’ usage behavior [21, 62]. Based on the above findings, this study took a further step and proved that perceived usefulness and users’ habits significantly affect users’ satisfaction, both of which had a positive effect on bike sharing users’ continued usage intention. The reason why the effect of perceived ease of use on users’ satisfaction was not significant may be that bike sharing users’ satisfaction is mainly affected by users’ perceptions of usefulness. This result is consistent with Amin, et al. [63] who asserted that users exhibit higher degrees of satisfaction for perceived usefulness than perceived ease of use.

Second, SEM results showed that service quality had a positive effect on users’ satisfaction, which positively influenced the decisions of riders to continue with the bike sharing service. However, the effect of system quality on bike sharing users’ continues usage intention was not significant. Prior research showed that service quality could affect users’ satisfaction and loyalty in the marketing area [64]. This paper found that service quality could affect users’ satisfaction, which subsequently influenced users’ continued usage intentions. The effect of system quality on users’ satisfaction was not significant perhaps because the quality of the bike sharing system is high [4] and its impact on users’ satisfaction is relatively small.

Third, SEM results showed that weather had a direct effect on shared bike users’ intentions, while the effect of travel distance was not significant. Previous studies showed that weather was an important factor in determining riders’ behavior [7, 41]. This study found that weather directly affected people’s desire to travel by means of shared bikes. Campbell, et al. [1] found that trip distance, temperature, precipitation, and poor air quality negatively impacted bike share demand. However, this study found no evidence that travel distance affected riders’ usage intentions, perhaps because bike sharing demand may limit users’ intentions.

Fourth, fsQCA results showed that six combinations of the variables were sufficient to explain users’ continued usage intention. Specifically, the most important combinations of conditions were service quality × perceived usefulness × perceived ease of use × weather × satisfaction and system quality × perceived usefulness × perceived ease of use × habit × weather × satisfaction because they have the highest raw coverage values. This conclusion was basically consistent with the results of the structural equation model, which meant that users’ perceptions of usefulness, habit, satisfaction, and service quality and weather were necessary for users of bike-sharing to resolve to stay with the service.

### Theoretical and practical implications

This study has important theoretical and practical implications. Firstly, we contributed new data to the understanding of the post-acceptance IS continuance model by evaluating the factors affecting bike sharing users’ continuing usage intentions. We examined how factors personal to users, inherent in the system, weather and trip distance all affected the decisions of riders to continue bike sharing. Previously published research on bike-sharing mainly focused on factors influencing the choice of bicycles [1, 2], the shared bike user’s subjective wellbeing [4], the diffusion of public bike sharing systems [5], and bike sharing’s impact on other types of transportation such as private cars [6]. Few studies have examined what aspects of the service most influenced the decisions of riders to stop or continue using bike-sharing. This study found that perceived usefulness, habits and satisfaction, service quality and weather were the most important factors.

Secondly, we contributed to the shared-bike literature by using two analytical methods. This paper provides an additional configural nuance in our understanding of the determinants of remixing that could have been only very partially intuited based on simple regression analyses. Most prior studies provided a good understanding of the underlying mechanisms of shared bike use [3, 16], however, few studies have focused on the configural nuance and effective path of bike sharing users’ intentions to continue. This paper further contributes to the existing data base by exploring six combinations of variables that are sufficient conditions for explaining users’ continued usage intention. Specifically, how useful the users perceived the service to be to them, how well it fit with their personal habits, their overall feelings of satisfaction, and the quality of service and influence of weather were all necessary for bike-sharing users to achieve high continuance levels. The results deepen our understanding about factors affecting bike-share use.

Finally, there are a number of practical implications that can be derived from this study. Bike-share operators may obtain some insights from the results of this research that could help them to strengthen their competitiveness. According to our SEM and fsQCA results, feelings of usefulness, agreement with user habits, satisfaction with good quality service and positive influence of weather were all factors combining to support a rider’s decision to persist in using bike-sharing. Thus, the operators of shared bicycle services should consider these factors when determining the next steps in their effort to popularize this mode of bike travel. For example, shared bicycle operators could issue coupons to enable users to form travel habits, improve service quality, strengthen the system’s user-friendly design to improve satisfaction, and make riding more convenient by enhancing the usefulness of the bicycle design.

### Limitations and future research

This research has several limitations that suggest directions for future research. Firstly, this study only considered user factors, system factors, and environment factors in affecting riders’ continued intentions to use shared bikes. We didn’t consider other factors, such as the supply of shared bikes, which may influence intentions. Secondly, the influence of weather and travel distance was assessed by single questions, which may need more expansion in future studies. Finally, this research used the questionnaire method to examine the effects of subjective psychological factors on users’ willingness to continue using bike-sharing services. Future investigations should also evaluate the attitudes of willingness and behavior of bike sharing users through a combination of cross-sectional and longitudinal research.

## Supporting information

S1 File (RAR)
